# Refractory Ventricle Arrhythmias Alternating with Pulseless Electrical Activity in a Young Woman Rescued by Extracorporeal Cardiopulmonary Resuscitation

**DOI:** 10.1155/2018/5686790

**Published:** 2018-01-14

**Authors:** Ilona Lálová, Lucie Filipovská, Hana Skalická, Ondřej Šmíd, Aleš Linhart, Helena Kollárová, Jan Bělohlávek

**Affiliations:** ^1^2nd Department of Medicine–Department of Cardiovascular Medicine, First Faculty of Medicine, Charles University in Prague, General University Hospital in Prague, U Nemocnice 2, 128 00 Prague 2, Czech Republic; ^2^Emergency Medical Service Prague, Korunni 98, 101 00 Prague 10, Czech Republic; ^3^Department of Preventive Medicine, Faculty of Medicine and Dentistry, Palacký University Olomouc, Olomouc, Czech Republic

## Abstract

**Introduction:**

Extracorporeal cardiopulmonary resuscitation (ECPR) is a challenging approach for treating refractory out-of-hospital cardiac arrest (OHCA).

**Case Presentation:**

The authors describe a case of a 40-year-old Caucasian female who suffered from refractory OHCA, was admitted to a hospital while receiving ongoing cardiopulmonary resuscitation, and was connected to venoarterial extracorporeal membrane oxygenation 73 minutes after collapse. Ventricular tachyarrhythmias alternating with pulseless electrical activity resolved after eight hours. Following complete cardiac and neurological recovery, only adenoviral genome was found in myocardial biopsy. After 11 months, another episode of identical arrhythmias was rescued by an implantable cardioverter-defibrillator.

**Conclusion:**

Adequate prehospital and early hospital logistics is a prerequisite for successfully implementing extracorporeal cardiopulmonary resuscitation for refractory OHCA.

## 1. Introduction

The chances of surviving after cardiac arrest are generally poor. Worldwide, the neurologically favorable survival rate in patients resuscitated for out-of-hospital cardiac arrest (OHCA) is only 2–11%, increasing to 12–19% in patients with initially shockable rhythms [1] and up to 22% for patients whose cardiac arrest occurs in-hospital [2]. In certain subgroups of patients with witnessed cardiac arrest, initial shockable rhythms, high-quality post-CPR (cardiopulmonary resuscitation) care with target temperature management and early coronary reperfusion can reach neurologically favorable outcome as much as 25–30% [1].

The main aim of the initial CPR is to attain the return of spontaneous circulation (ROSC), as prolonged cardiac arrest (CA) has dismal prognosis [3]. In patients without ROSC, the chance of survival after being transported to a hospital under ongoing CPR is very low, that is, less than 4% [4, 5]. Therefore, in selected cases of OHCA, substituting failed spontaneous circulation by an extracorporeal device seems to be an attractive approach, that is, to use extracorporeal cardiopulmonary resuscitation (ECPR).

ECPR has been recognized in the European Resuscitation Guidelines 2015 as a rescue therapy for those patients in whom initial advanced life-support measures are unsuccessful. ECPR can also facilitate specific interventions such as coronary angiography and percutaneous coronary intervention (PCI) or pulmonary thrombectomy for massive pulmonary embolism [6]. The American Heart Association (AHA) guidelines are more cautious regarding the recommendation of ECPR because of the ongoing paucity of available data [7]. A current meta-analysis compared ECPR to conventional CPR and proved a favorable effect of ECPR on a 3- to 6-month survival and good neurological outcome in ECPR. However, the effect of ECPR on survival to discharge in OHCA was not clearly shown, indicating a need for strict criteria for implementing [8].

The presented case aims to demonstrate a successful prehospital and early hospital logistics for a patient with prolonged refractory OHCA without ROSC who was transported to a hospital under ongoing CPR and received emergency ECPR resulting in a favorable outcome.

## 2. Case Report

**Figure 1:**
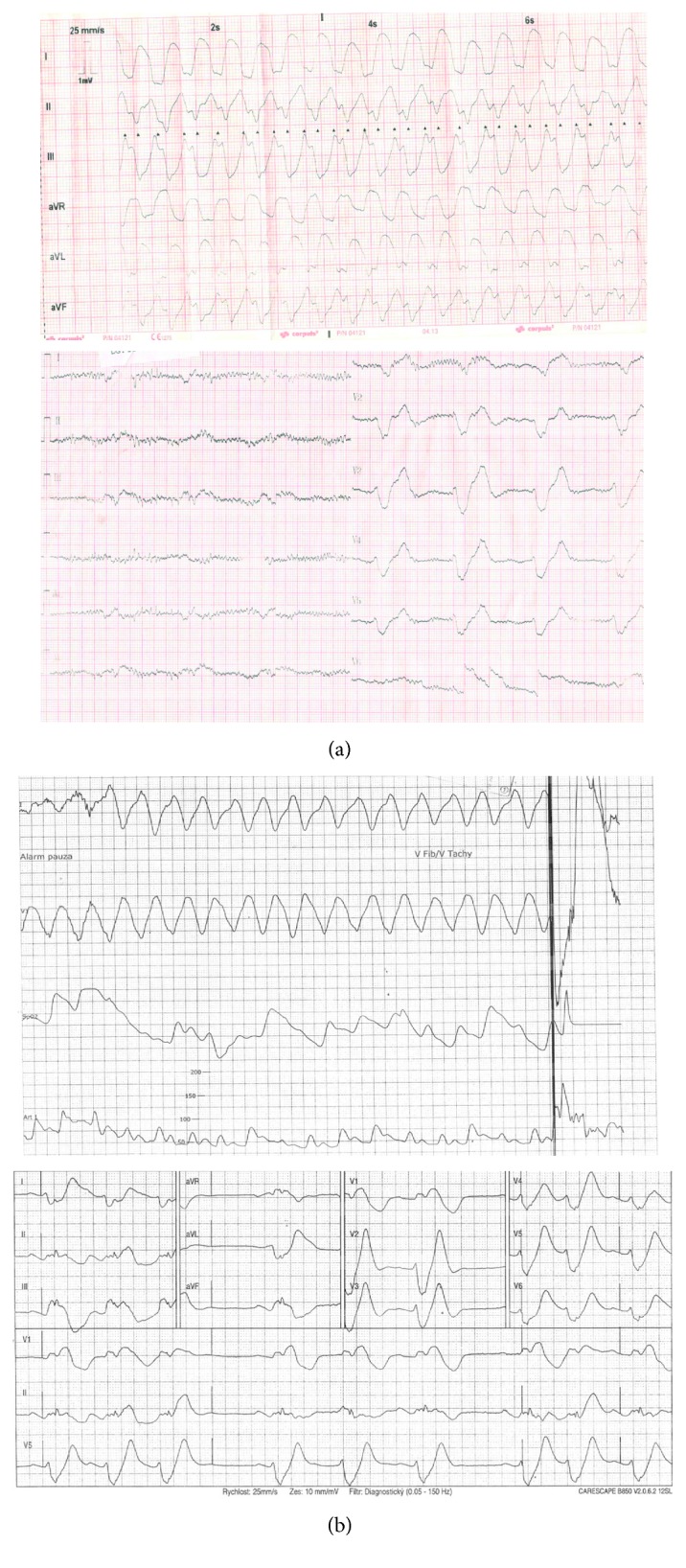
ECG tracings during the index episode of prolonged cardiac arrest (a) and almost a year later (b).

This case report describes the events following a refractory cardiac arrest in a 40-year-old Caucasian female with a history of atypical chest pain and vertigo. She first collapsed when walking with her family members. While waiting for emergency medical service (EMS) to arrive, she regained full consciousness. However, another three short episodes of a loss of consciousness reoccurred. Shortly after the EMS team arrived, she collapsed again, ventricle fibrillation (VF) was detected on electrocardiography, and immediate CPR was initiated. After 26 minutes of manual CPR with an ongoing refractory VF (6 unsuccessful defibrillations), the patient was switched to mechanical CPR with a LUCAS device (Lund University Cardiac Arrest System; Physio-Control Inc./Jolife AB, Lund, Sweden) and transported to hospital under ongoing CPR, which was carried out after alerting the cardiac center. Upon arrival to the catheterization lab, the patient was unconscious with wide mydriasis and without palpable pulses. The ECG tracings showed complex ventricle tachyarrhythmias alternating with wide complex bradycardia (Figure 1).

Laboratory values on admission showed a pH of 7.05, lactate of 12 mmol/L, and brain regional tissue saturations measured by near-infrared spectroscopy of 19 and 15% in the right and left hemispheres, respectively. The patient was urgently implanted with peripheral venoarterial ECMO using a femorofemoral approach. Cannulation lasted 16 minutes after being admitted to the cathlab, and the overall cardiac arrest time until extracorporeal circulation was 73 minutes. Next, coronary angiography and pulmonary angiography were performed with completely normal findings. The patient was then transferred to the intensive care unit while several types of arrhythmias (VF, sustained ventricle tachycardias, and wide complex bradycardia) were still ongoing. After six more failed defibrillations, no more shocks were provided. Eight hours after cardiac arrest, intravenous esmolol and amiodarone stabilized the heart rhythm to a sinus. The core body temperature was maintained at 36°C for 12 hours. The next day, the patient was extubated to full consciousness without any neurological sequelae, and ECMO was explanted 3 days later. The patient had undergone a thorough search for the cause of cardiac arrest including echocardiography and cardiac magnetic resonance imaging but displayed completely normal findings. The toxicology report was also negative, and a myocardial biopsy revealed adenoviral genome as the only potential cause of the cardiac arrest. A laboratory examination for possible channelopathies was also completely negative. An implantable cardioverter-defibrillator (ICD) was implanted, and the patient was discharged after 30 days of hospitalization. Eleven months later, a similar episode of ventricle tachycardias alternating with wide complex bradycardias occurred again and was rescued by the ICD (Figure 1). Unfortunately, the etiology of this second episode still remained unclear. To date, three years after the initial cardiac arrest, the patient is doing well and has returned to work without any further adverse events.

## 3. Discussion

This case illustrates the need for a structured approach for both prehospital and early hospital logistics. The patient was quickly identified as a case of refractory ventricle fibrillation, a mechanical CPR device was promptly started, and transport under ongoing CPR was carried out enroute to a cooperative ECPR cardiac arrest center. Additionally, the fact of complete neurological recovery highlights the potential of ECPR to rescue a patient with prolonged cardiac arrest. The patient was actually an optimal candidate for ECPR. Cardiac arrest occurred while the emergency medical team was on the scene, the initial rhythm was shockable, the patient was young, immediate high-quality CPR was started, and early switch to transport under CPR to hospital was undertaken after 6 unsuccessful defibrillations.

It is controversial how long to wait until decision to transport under CPR is done. It seems reasonable to wait at least 10–15 minutes after advanced life support is started by an emergency medical service/CPR team [3, 9]. As the waiting time increases up to 30 minutes, the chance of survival substantially decreases [10]. The final decision on whether to proceed with ECPR should be undertaken after an ECMO team decides and should be based on previously stated rules [11]. In the meantime, the ECMO team should be prepared in the cathlab or emergency department to immediately proceed with ECPR if cardiac arrest still persists on admission and the patient fulfils the criteria for ECMO implantation. In cooperation with the physician-stuffed prehospital emergency medical service [12], we use a structured approach based on previously published protocol both for enrollment of the patient into the “ECPR” track and also for the final decision whether to connect an ECMO [11]. Inappropriate connection to ECMO may portend a risk of serious consequences for both the patient and his relatives [13].

This case is unique not only because the patient survived an exceptionally long period of CPR before ECPR was launched (73 minutes of resuscitated cardiac arrest) but also because of an additional eight hours of ongoing VF while on ECMO with subsequent complete cardiac recovery. Moreover, the cause of cardiac arrest was not determined despite detailed examination. Adenoviral genome detected by myocardial biopsy was the only potential cause, as all other examinations including coronary angiography, echocardiography, and cardiac magnetic resonance imaging were completely normal. We are not entirely sure whether this adenoviral infection might have caused the refractory cardiac arrest, but fatal cases and impaired left ventricular function have been reported [14–16]. Interestingly, almost a year later, the patient had a very similar episode. This time, probably due to early ICD discharges, the patient did not suffer a cardiac arrest and survived the second episode of the arrhythmogenic storm.

## 4. Conclusion

This case illustrates an optimal scenario for treating refractory OHCA, which includes reasonable time on scene to try to attain ROSC, if not, transport to the hospital under ongoing CPR followed by an immediate implantation of ECMO, that is, the ECPR. Despite a CPR time of 73 minutes and VF continuing for 8 hours, the patient survived with a favorable neurological outcome. In selected cases, implementing ECPR into routine care for refractory OHCA seems reasonable, and all efforts should be undertaken to adjust prehospital and early hospital logistics to provide emergency ECPR in such scenarios.
